# Design and Experimental Characterization of a Discovery and Tracking System for Optical Camera Communications †

**DOI:** 10.3390/s21092925

**Published:** 2021-04-22

**Authors:** Antonio Mederos-Barrera, Cristo Jurado-Verdu, Victor Guerra, Jose Rabadan, Rafael Perez-Jimenez

**Affiliations:** Institute for Technological Development and Innovation in Communications (IDeTIC), Universidad de Las Palmas de Gran Canaria (ULPGC), 35017 Las Palmas de Gran Canaria, Spain; cjurado@idetic.eu (C.J.-V.); vguerra@idetic.eu (V.G.); jose.rabadan@ulpgc.es (J.R.); rafael.perez@ulpgc.es (R.P.-J.)

**Keywords:** optical camera communications, detection system, tracking system, discovery system, test system for tracking systems, K parameters

## Abstract

Visible light communications (VLC) technology is emerging as a candidate to meet the demand for interconnected devices’ communications. However, the costs of incorporating specific hardware into end-user devices slow down its market entry. Optical camera communication (OCC) technology paves the way by reusing cameras as receivers. These systems have generally been evaluated under static conditions, in which transmitting sources are recognized using computationally expensive discovery algorithms. In vehicle-to-vehicle networks and wearable devices, tracking algorithms, as proposed in this work, allow one to reduce the time required to locate a moving source and hence the latency of these systems, increasing the data rate by up to 2100%. The proposed receiver architecture combines discovery and tracking algorithms that analyze spatial features of a custom RGB LED transmitter matrix, highlighted in the scene by varying the cameras’ exposure time. By using an anchor LED and changing the intensity of the green LED, the receiver can track the light source with a slow temporal deterioration. Moreover, data bits sent over the red and blue channels do not significantly affect detection, hence transmission occurs uninterrupted. Finally, a novel experimental methodology to evaluate the evolution of the detection’s performance is proposed. With the analysis of the mean and standard deviation of novel K parameters, it is possible to evaluate the detected region-of-interest scale and centrality against the transmitter source’s ideal location.

## 1. Introduction

The number of interconnected devices has increased considerably in recent decades [1]. Connected devices are limited to those used by humans, such as cell phones, but to autonomous ones, which operate without human interventions. Furthermore, wireless sensor network and Internet of Things technologies [1] allow small electronic equipment to have telecommunication systems. The growth of interconnected devices has led to a patent saturation of the spectrum, in which the intelligent allocation of frequencies faces more and more difficulties in its performance.

Wireless optical communications (OWC) [2] are currently positioned as a set of technologies that can reduce the saturation of the radio spectrum by using the visible and near-infrared spectrum, considering a suitable candidate as a communications support infrastructure in the new 6G networks [3].

Visible light communications (VLC) [4,5] cover a field of OWC, which aims to reuse LED light luminaries present in existing illumination infrastructures as optical transmitters. Despite its promising capabilities, commercial products using VLC technology currently have challenging market entry barriers due to the need to incorporate specific hardware into end-user devices. As a solution to this problem, a new branch of VLC is proposed, known as optical camera communication (OCC), which uses cameras as optical receivers [6,7,8]. Additionally, the IEEE 802.15.7r1 task group is responsible for establishing a standard to accommodate the visible, infrared, and near-ultraviolet wavelengths within wireless personal area networks (WPAN), providing OCC as a technological and commercial solution that enables scalable data transmissions, broadcasting, and positioning, among other applications [9].

**Figure 1:**
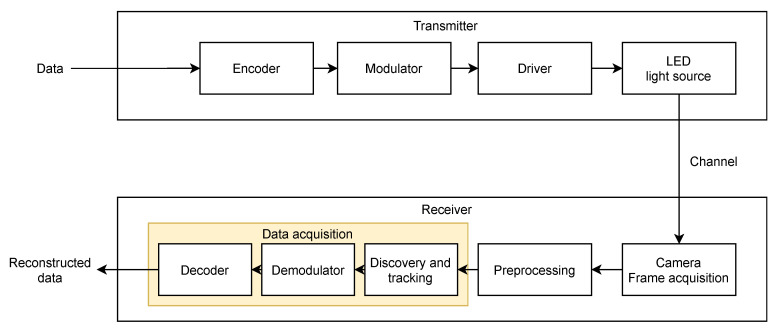
Diagram of an OCC system.

Regarding the transmitters, OCC sources have certain similarities with those of VLC, such as the use of a driver. However, they differ mainly in the communication strategies used and the size of the lighting surface area that tends to be higher to increase the transmission rate. Figure 1 shows the general diagram of the transmitter of an OCC system, composed of the encoder, modulator, LED driver, and LED source.

Regarding the receiver, Figure 1 shows the general scheme, composed of the camera, the preprocessing system, and the data acquisition system, consisting of the demodulator and the decoder.

One of the main challenges of OCC is to efficiently use the low bandwidth offered by the cameras to increase the bit rate. OCC systems are limited by the rate of frames per second (FPS) of cameras. Higher FPS, the greater the number of images per second, increases the capacity of the system. Since conventional cameras are limited in FPS, the potential applications are those requiring low bit rate transmission, like in the field of Internet of Things (IoT) [10]: localization [11], vehicle-to-vehicle (V2V) communications [12], or underwater communications [13], among others.

To increase the bit rate of OCC systems, cameras with a higher FPS capture rate are chosen. However, the cost increases considerably. Therefore, other techniques are needed to increase the bit rate in order to have a commercial OCC solution.

In OCC, two types of cameras can be used, global shutter (GS) and rolling shutter (RS) cameras. In GS cameras, all the rows of the image sensor are captured simultaneously. In this type of camera, undersampled modulations can be used to reduce the flickering and increase data throughput [14], such as undersampled frequency shift ON-OFF keying (UFSOOK) [15], undersampled phase shift ON-OFF keying (UPSOOK) [16], or undersampled color shift keying (UCSK) [17].

On the other hand, in RS cameras, the images are captured line by line. Using ON-OFF Keying (OOK) modulation, and choosing a symbol transmission time close to the row shift time (the time that elapses between the start to two consecutive rows), intensity bands are formed in the captured image [18]. Each band corresponds to one symbol transmitted in the case of using OOK modulation. Therefore, the use of OOK modulation can obtain higher bit rates in RS cameras than in GS cameras.

Regarding error detection and correction, other techniques can be used, such as using low-density parity check (LDPC) codes [19,20] or turbo-like decoder to improve the system throughput at the cost of increasing complexity [21,22].

Another way to increase the binary rates is spatial multiplexing, that is, positioning different information at separate image points. This strategy was used in combination with RS cameras in [23], using a matrix of LEDs grouped into vertical bars. This allows the number of LEDs to be reduced, enabling them to be replaced by bars that are fully utilized to send data. However, because the data are not synchronized within the bars, it is difficult to establish synchronization mechanisms, such as anchor beacons, to facilitate source detection. In this article, a LED matrix with fixed position beacons is proposed, which uses GS strategies instead for demodulation.

However, the increase of data rate is not the only challenge in those systems. Another major challenge is the real-time detection of the source within the image. This functionality is usually implemented using a discovery block, as shown in Figure 1.

The discovery block performs the detection of the transmitting sources in the image, considered the region of interest (ROI). This region needs to be estimated for each incoming image and provided to the subsequent blocks; the demodulator and decoder.

In this type of architecture, the discovery block becomes a bottleneck quickly in terms of processing speed. The times invested in detecting the source for each frame considerably slow down data acquisition rates, which in the worst cases, can cause unacceptable latencies for systems that need real-time communications. Therefore, it is necessary to add a tracking block (Figure 1), which, in conjunction with the discovery algorithms, improves the source’s detection times. This tracking block would reduce the overall detection time because it only evaluates slight interframe differences in regions near the source’s previous positions and not in the entire frame. This tracking algorithm should respond quickly so that dynamic communications are allowed, where the emitter and the receiver can move freely in the scene at different speeds. This work’s main objective is to evaluate the performance of this proposed architecture in which the discovery block finds the transmitting source with high precision, and the tracking block follows it over time.

Finally, a novel evaluation method based on statistical parameters is also presented to measure the discovery and tracking performance and progression over time.

This document is structured as follows: in Section 2, the existing system and the proposed system are presented; in Section 3, the materials and methods are presented, where the test system is proposed, to observe the performance of the system, and the experiment setup is exposed; in Section 4, the results obtained from the test system are shown and discussed; finally, in Section 5, the article is concluded.

## 2. Proposed System

In this section, the transmitting source is presented, and the proposed receiver architecture for the receiver is detailed and compared to the state of the art. Both discovery and tracking algorithms are discussed.

### 2.1. Transmitter

The transmitting source consists of a matrix arrangement of spot RGB LEDs equispaced in Cartesian coordinates. This structure efficiently exploits the spatial multiplexing capabilities of the cameras. In [23], a similar matrix is configured as a transmitter source. The main difference is that the transmitter is designed to operate with rolling shutter cameras that scan the image line by line of pixels. In this work, even though a rolling shutter camera is used, the symbol time is greater than the frame acquisition time, so it can be considered that the system implements global shutter strategies. That is, only one symbol per image can be retrieved for each LED. Another difference is that this system adds detection mechanisms (such as anchor LEDs) to address the relative movement between the source and the camera.

In the LED matrix, the green channel is always kept on to make it easier for the receiver to discover and track the source. This allows the transmission to take place in the rest of the channels without significantly deteriorating the spatial characteristics of the source analyzed by the source detection algorithms. Therefore, communications can occur concurrently without significantly affecting source detection.

The other color channels (red and blue) are left for data transmission with OOK modulation. However, the proposed system does not emphasize the selection of a specific modulation mechanism, and the one that is most convenient for each case can be chosen.

The reason behind selecting the green channel as the detection channel is because it generates similar interference on adjacent channels, which can be successfully subtracted in the final decoding of the data. Moreover, this configuration allows the OFF values for the data channels to be set to zero intensity value, increasing the difference between ON and OFF states, thus improving the signal noise ratio.

On the other hand, to measure the channel interferences between the channels at reception, four training LEDs are placed at the edges of the matrix. These LEDs emit a combination of different light colors, resulting in output colors of white (red, green, and blue), yellow (red and green), cyan (green and blue), and green.

Before decoding, the transmitter orientation must be established. Three anchor LEDs, which form a scalene right triangle, are selected from the training LEDs at three corners of the array. In this case, the upper right, lower left, and lower right corners are chosen. This figure is used since the angle of rotation can be obtained unequivocally. If the figure is rotated, there is only one rotation angle where the rotated image matches the original image, unlike other figures such as the rectangle with four coincidence angles.

**Figure 2:**
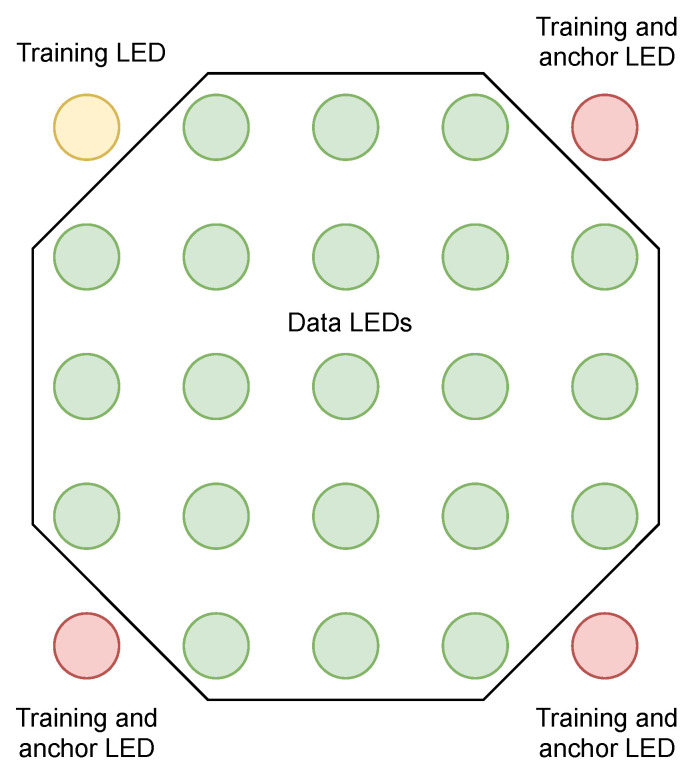
Transmitter LED data structure.

The schematic of the transmitter structure is presented in Figure 2.

Regarding the synchronization of the modulation data, it is decided to keep the data transmission twice as long as the capture, the inverse of the FPS. Therefore, one frame is discarded for every two to avoid transitioning effects. The only training LED, which is not an anchor LED, is used to detect the transition frame. To detect the transition, this LED varies its intensity level between two values. It is important to highlight that the system uses a global shutter camera.

The bit rate, $B_{r}$, limit is given by the expression (1).

(1) $$B_{r}=\left(2\;\cdot \;N\;\cdot \;M-8\right)\;\cdot \;FPS/2$$

where *N*, and *M* are the number of rows and columns of the LED matrix.

**Figure 3:**
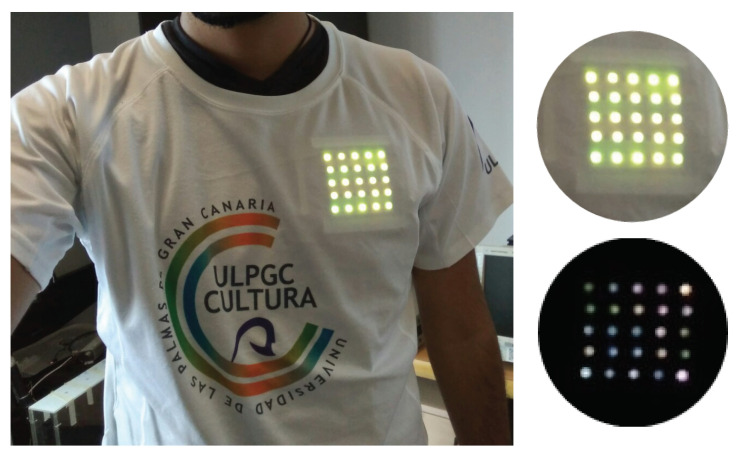
Transmitter implementation.

The expected use case of this transmitter is related to wearable devices. In Figure 3, an example of the transmitter placed behind a T-shirt is shown. In addition, on the right side of the image, the transmitter’s effect is observed by lowering the exposure time.

### 2.2. Receiver

Nowadays, OCC receivers operate mainly in static conditions, where the relative movement between the source and the receiver is small. In these conditions, they must discover the source in the image from time to time, when either too many errors occur during the transmission or regularly to ensure the link operation. However, when this movement exceeds minimum motionless criteria, the discovery algorithms must continuously be used either over the total or fraction of the image. In addition, discovery algorithms do not differentiate between an initial detection case and a follow-up case. They behave in the same way, which entails considerably high detection times that slow down the data acquisition.

Unambiguous source discovery relies on certain source features, either in the spatial or the temporal domain. In the spatial domain, differences in shape, relative size in image, or color are utilized. On the other hand, in the temporal domain, the variation between two consecutive frames of various parameters, such as brightness or hue, is examined.

Although both strategies can be used to locate the source, to recognize temporal variations, the transmitter must send a beacon signal over time. This beacon must be sent periodically to facilitate source detection and tracking, which considerably reduces the time available for effective communication. The design of this beacon must be carefully adapted to the coding strategies used to be recognizable and distinguishable from data transmission. To avoid data interruptions, ref. [24] proposes a new hybrid waveform that combines two OCC signals. One faster for data transmission, and the other, for ROI signaling. This system solves the problem of interrupted communication associated with evaluating temporal features for detection at the expense of implementing two OCC receivers that operate concurrently. Furthermore, it implies an indirect coupling between the coding and detection strategies when designing the hybrid signal.

In addition, during beacon detection, the sources must be precisely located between consecutive frames. Therefore, the source should move in slow relative motion, or the receiver might use complex algorithms to predict and correct the source’s movement. To ensure these static conditions, ref. [25] uses a high-speed camera with over 1000 FPS to detect traffic lights. To perform the detection, it subtracts consecutive frames to enhance the transmitter sources. The misalignment between both frames can cause unwanted errors in the detection and depend on the camera’s capture speed, the relative source’s speed, and distance.

On the other hand, spatial detection uses object detection algorithms to discover the transmitter’s position in the frame, based on the source’s features (such as shape, color, or light intensity). This allows communication to proceed without temporal interruption. The fact that the legitimate sources’ spatial characteristics are analyzed allows the discovery strategies to be separated from the data transmission. Thus, the detection and transmission can happen concurrently. In this case, the receiver can detect the source and decode it using a few consecutive frames. In [26], convolutional neural networks (CNN) are implemented to discriminate the transmitting source from unwanted active optical objects, such as streetlights in the case of vehicle-to-vehicle communications, with an average precision of 60% in natural night conditions, with an elapsed time of 13 s, approximately.

### 2.3. Discovery and Tracking Proposed Architecture

As mentioned previously, the reuse of discovery algorithms to track the source over time involves high computational costs. In this work, it is proposed to use tracking algorithms that follow the source more quickly, while ensuring its location. The discovery and tracking algorithm proposed in this work is based on spatial features, rather than temporal variations, to prevent data transmission interruptions. It uses conventional algorithms with an architecture optimized for OCC.

**Figure 4:**
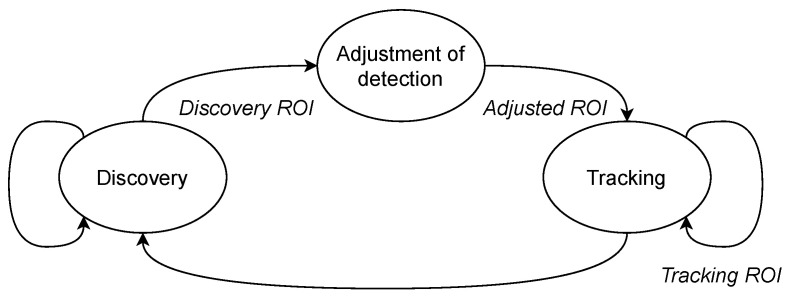
Diagram of the discovery and tracking proposed architecture.

The state diagram in which the receiving system is before the decoding stage is shown in Figure 4. Initially, the system discovers the ROI within the first image containing the transmitter. For future references, this ROI would be identified as discovery ROI. If the source is not detected, the system returns to the discovery state successfully. This process is performed until the source is discovered when the receiver enters a tracking state. For tracking initialization, the system adapts the discovery ROI slightly and provides it to the tracking algorithm. This initialization ROI is called selected ROI. As new images are acquired, the receiver will attempt to locate the image’s source location based on the previously stored position using the tracking algorithms. The ROI delivered by the tracking algorithms is known as tracking ROI.

#### 2.3.1. Discovery Algorithm

The discovery algorithms will return the location of the transmitting source in the image. For this purpose, these algorithms use spatial features of the source. To reduce the computation time, the whole image is not entirely evaluated, but some image’s regions called proposals. Proposals are regions of the image where it is very likely that the transmitter source is found. Initially, the system generates a list of proposals for later classification as belonging to a legitimate transmitting source. To facilitate the generation of the proposals, the exposure time is reduced, making it easier for transmitting sources to stand out in the image against other objects. Therefore, the number of proposals to be classified is reduced, reducing the time execution and increasing the overall processing FPS. The use of generation of proposals is present in models such as R-CNN [27], Fast-RCNN [28], Faster-RCNN [29] or YOLO [30].

Two techniques are considered for the generation of proposals: selective search [31] and edge boxes [32]. In selective search, the image is initially over-segmented. Segments with the same characteristics are joined by the similarity of color, texture, size, and shape. The ROI that encloses the segment is the proposal. On the other hand, edge boxes are based on the fact that the number of contours enclosing a region is proportional to the probability that an object exists within the region. Initially, an edge map is obtained. Edge groups are generated on the map. Finally, proposals are generated from the groups with the highest proportion of edges.

#### 2.3.2. Tracking Algorithm

The use of tracking algorithms reduces computation time considerably, since it does not evaluate the entire image, but the regions local to the positions where the source was previously located. Therefore, this algorithm returns an ROI where it is very likely that the source will be found.

As time passes and the source moves, the source’s location in the image, proposed by the algorithm, tends to differ from the actual location. Either the returned ROI decentralizes or expands and contracts over time. Therefore, it is necessary to return periodically to an initial state of discovery to recover the accurate position.

Tracking algorithms apply two different techniques in their operation: motion or appearance analysis. In motion-based algorithms, depending on the object’s speed and direction in previous frames, a rough estimate of the object’s position in the current frame is made. On the other hand, in appearance-based algorithms, studying the object’s features (such as color or edges), the fine adjustment is performed concerning the motion model’s coarse adjustment.

The tracking algorithm considered in this works is shown in Table 1.

In Section 4, the performance of the different tracking algorithms is presented. From this analysis, the best algorithm is selected based on the established metrics.

#### 2.3.3. Adjustment of Detection

To facilitate the tracking of the transmitting source, the tracking algorithm is initialized with an ROI that is slightly greater than the ROI delivered by the discovery algorithm, the selected ROI. This ROI is therefore extended towards the edges by a delta factor, specified in pixels. This expansion allows the tracking algorithm to correct minor deviations from source centrality and adapt more precisely to changes. Moreover, it ensures that the source remains for a longer duration within the ROI, thus considerably increasing the return-to-discovery times. However, this expansion cannot increase indefinitely. Otherwise, other light emitters falling within the ROI would interfere, causing the desired source to be lost track quickly.

**Figure 5:**
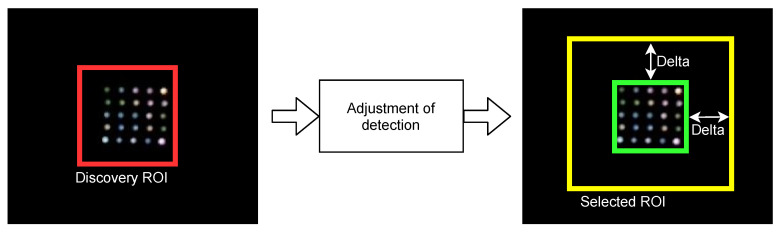
Example of operation of the adjustment of detection.

Figure 5 shows an example of the adjustment of detection. On the left, the discovery ROI, which contains the transmitter, is shown in red. It can be seen that the initial ROI is decentralized and does not have a proper scale. On the other hand, once the detection setting has been applied, the centralized and scaled ROI is shown in green on the right side. Finally, the yellow rectangle encloses the selected ROI with the delta margin.

## 3. Materials and Methods

In this chapter, the methods and metrics used in evaluating the proposed discovery and tracking algorithms performance and the experiment setup are presented and detailed.

### 3.1. Methods and Metrics

The proposed experiment to evaluate the discovery and tracking algorithms is divided into two sequential phases: the recording phase and the processing or evaluation phase.

In the first phase, a transmitting source moving at constant speeds is recorded. A constant velocity is preferred to evaluate the system over time. Highlight that during recording, the system is transmitting pseudo-random data using PRNG (pseudorandom number generator) algorithms. This allows the performance of the detection algorithms to be evaluated independently of the transmitted data. In addition, the experiment is recorded within indoor laboratory conditions.

In the second phase, both the discovery and tracking algorithm are evaluated separately. The metrics discussed at the end of the chapter are computed to evaluate the performance of the discovery and tracking algorithms. Highlight that the tracking algorithm’s analysis relies on the results obtained during the discovery algorithm’s evaluation.

For both discovery and tracking, three types of movements are analyzed: lateral, diagonal, and frontal. In lateral movement, the transmitter moves in the perpendicular plane concerning the camera, with 0° of inclination. In the diagonal movement, the transmitter also moves in the perpendicular plane, but with 45° of inclination. Finally, in the frontal movement, the transmitter moves in the central axis normal to the camera. The transmitter moves away from and towards the camera.

Regarding the metrics used for evaluating the performance of discovery algorithm, the following are contemplated: average execution time and recall. The latter parameter measures the system’s ability not to discard legitimate sources in the image. The definition of recall is given in Equation (2).

(2) $$Recall=\frac{True_{positives}}{\left(True_{positives}+False_{negatives}\right)}$$

where $True_{positives}$ cases are those where the receiver detects a legitimate source in the image correctly, and $False_{negative}$ are those cases in which the system misses a legitimate source.

A preliminary analysis of the proposal generation algorithms reveals that the Selective Search has a computation time of 10 s greater than the edge boxes ( 0.2 s), for the same image. However, both algorithms have approximately the same recall (87%). Therefore, the edge boxes algorithm is preferred for the implementation of the detection system. On the other hand, for the proposals classification, an algorithm is chosen that detects and lists the contours within the evaluation region using binarization and edge detection.

Regarding the tracking evaluation, preliminary metrics are considered: average execution time and scalability. The last parameter refers to the system’s ability to track sources that move closer and further away from the camera, increasing and decreasing the area of their projection on the image. Some current tracking mechanisms do not support that the object increases or decreases its size in the image. Therefore, they have to be discarded for the implementation of this system. In addition, this preliminary evaluation allows one to discard those algorithms that are less efficient.

After the preliminary evaluation of the tracking algorithms, the deterioration of the tracking ROI is analyzed. For this purpose, an initial ROI, obtained by the discovery algorithm, is selected, and the edges are slightly expanded (delta pixel basis). This ROI is delivered to the tracking algorithm as selected ROI. The ROIs that the tracking algorithm returns for a number *n* of consecutive frames are then stored. These ROIs are then compared against the ROIs that the discovery algorithm would deliver if used instead of the tracker algorithm.

**Figure 6:**
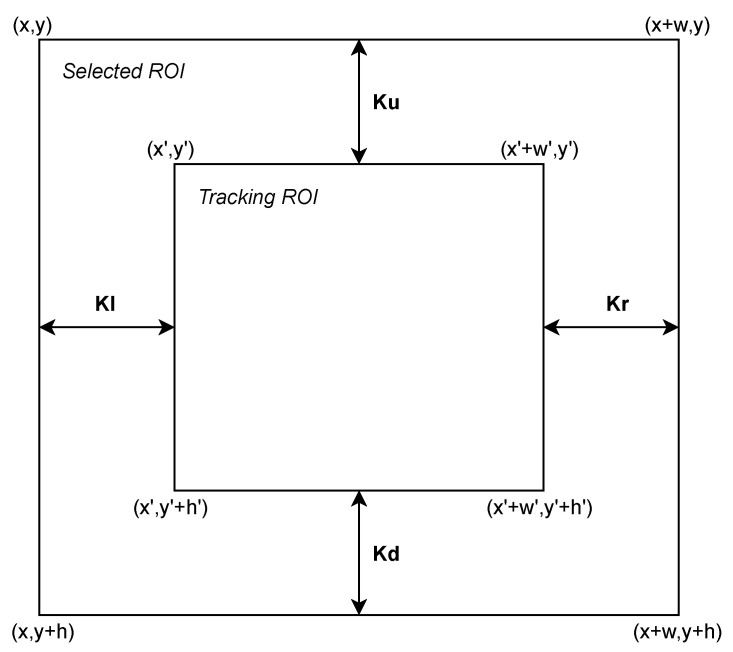
Representation of K parameters.

Under perfect conditions, the selected ROI and the tracking ROI should coincide to a large extent. It should stay centered and maintain its relative size of delta pixels on all edges. However, as was aforementioned, deterioration is accentuated over time. To evaluate this deterioration, the difference in distance between the edges of both ROIs it analyzed. These distances are identified as K parameters shown in Figure 6, which coincide with the upper, lower, left, and right separation (in pixels) of each ROI. These parameters are summarized in Equation (3).

(3) $$\begin{array}{cc} \; & K_{u}=y^{\prime }-y\\ \; & K_{d}=\left(h^{\prime }-h\right)-K_{u}\\ \; & K_{l}=x^{\prime }-x\\ \; & K_{r}=\left(w-w^{\prime }\right)-K_{l} \end{array}$$

The combined analysis of these parameters allows one to evaluate the centrality and scale deviation over time, using the mean and standard deviation of the overall K parameters.

The mean of K parameters indicates the scaling relationship between the tracking ROI and the selected ROI. In the case where the mean is zero, it indicates that the ROIs are equal in area. Otherwise, one ROI has a larger area than the other. In the case study, it is desired that the increase in area is constant over time and equal to the delta parameter. On the other hand, if the value is negative, part of the transmitter falls outside the tracking ROI. In this case, the transmitting source is considered to be lost.

The standard deviation of K parameters indicates the centrality. For values close to zero, centrality is preserved. Otherwise, it is shifted to any of the possible sides.

Finally, the temporal evolution of the scale and centrality is fitted using a linear regression. The slope and standard deviation of the residuals of this fitting illustrate the temporal trend and variability with respect to the trend, respectively, of the scalability and centrality over time.

However, since only a series of experiments have been carried out, it is necessary to contrast the values obtained for the means and variances using a Student’s *t*-test to determine if the regression has a linear behavior with a 95% confidence interval. The slope of the linear regression is studied. In this case, the null hypothesis is understood as a null slope, so the system converges to tracking. In this case, the system does not need to return to the discovery state. Otherwise, when the null hypothesis is rejected, the system must discover the source, since tracking will deteriorate over time.

**Figure 7:**
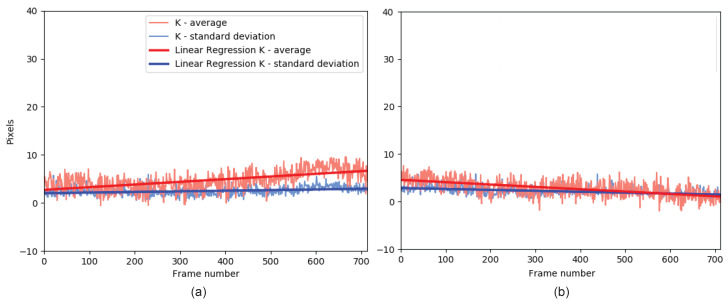
Example of the application of linear regression to K parameters: (**a**) example of positive slope, (**b**) example of negative slope.

Figure 7 shows two examples of positive slopes (Figure 7a) and negative slopes (Figure 7b). The red curve corresponds to the scale (mean of the K parameters), while the blue color corresponds to the variability (standard deviation of the K parameters).

A positive slope for the scale indicates that the tracking ROI area increases over time, while a negative slope indicates that the area decreases. Thus, a positive slope indicates that there is a higher probability of interference from an unwanted object. In contrast, a negative slope implies that the source is partially lost over time.

A positive slope for the centrality indicates that the tracking tends to decentralize, while a negative slope indicates the opposite. In this case, a positive slope indicates that the tracking ROI will have a more significant variation over time between consecutive frames, while a negative slope indicates that the tracking ROI will tend to remain static concerning the transmitter over time.

### 3.2. Experiment Setup

**Figure 8:**
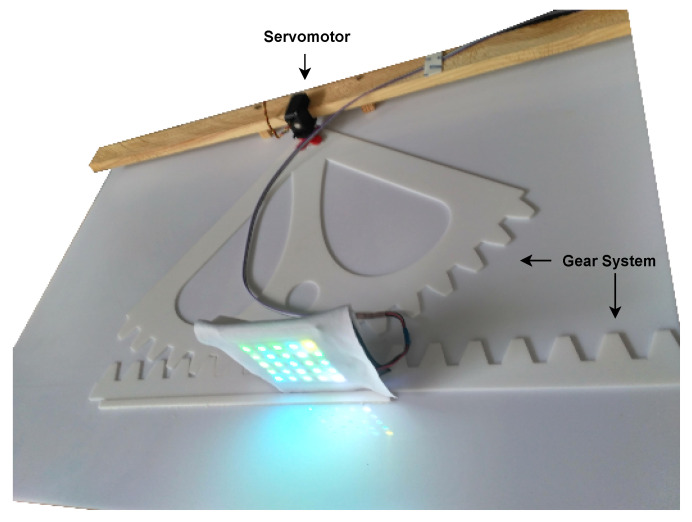
Mechanical system of test.

A system is designed to move the transmitter at a constant speed, shown in Figure 8. It consists of a servomotor, responsible for exerting the force to move the transmitter; a gear system, responsible for converting the motor’s torque into a uniform, straight motion; and a function generator, responsible for exciting the motor to move according to specifications. In the generator’s case, a triangular signal is generated to have a constant linear motion most of the time, except at the edges, where there are decelerations and accelerations.

The maximum achievable speed is approximately 1.1 m/s, imposed by the radius length of 0.35 m and rotor speed.

The experimental setup’s parameters and values are shown in Table 2.

## 4. Results

In this section, the results obtained are presented. As in the previous sections, the presentation of the results is divided into discovery and tracking algorithms.

### 4.1. Discovery Algorithm

Regarding the system’s recall, there are no significant differences in performance observed for the configuration parameters stated in Table 2. The value of recall is close to or equal to 99% in all cases. It can be concluded that as long as there is a direct vision of the transmitter and under the indoor conditions in which the experiment was conducted, the discovery will perfectly recover the transmitter’s location. Lowering the exposure time greatly improves discovery reducing the interference phenomena that hinder the detection of the transmitter. The proposal classification regarding if it belongs to a transmitter source has been carried out by examining if it contains 25 regular distributed contours. It can be established that the presence of other light sources in the image will not significantly affect the system’s accuracy in detecting legitimate sources. Regarding the execution time, it remains constant regardless of the capture frequency of the camera, as expected. The value obtained for the execution time is approximately 96 ms, considerably higher than the acquisition time of a frame. In terms of frequency, this implies that the system could only process images with a processing rate of $FPS=1/t_{execution}=10/11FPS$. Therefore, It can be concluded that under the conditions of the experiment, continuous discovery can be performed at 10 and 11 FPS, which considerably affects the maximum data transfer rate or increases the latency.

The use of edge boxes compared to the use of selective search allows one to considerably reduce the average execution time without decreasing the system’s recall, since it adapts very well to the nature of the image.

On the other hand, the presence of pseudo-random data in the payload does not affect source detection. This justifies the selection of beacons and the use of the cameras’ multiplexing capabilities for the detection of the transmitting sources.

### 4.2. Tracking Algorithm

The transmitter’s motion speed does not significantly affect the results obtained for the lateral and diagonal movements. The relative translation, in pixels, between two consecutive frames, it is relatively unlikely to observe differences in this aspect. There are two cases where this translation is the largest and the smallest. In the first case, the camera is located at a distance of 1.3 m and scans the transmitter moving with a speed of 1.1 m/s with a framerate of 90 FPS. The translation in pixels between two consecutive frames is about 7 pixels approximately. In the second case, the camera is located at a distance of 2 m and scans the transmitter moving with a speed of 0.5 m/s with a framerate of 60 FPS. In the latter case, the translation in pixels is about 2 pixels. To obtain these quantities, the Equation (4) is used.

(4) $$p_{r}=\frac{V_{T_{x}}\;\cdot \;res_{i}}{FPS\;\cdot \;2\;\cdot \;d_{T_{x}R_{x}}\;\cdot \;\tan\left(\frac{FoV_{i}}{2}\right)}$$

where $p_{r}$ is the relative translation between two consecutive frames in pixels, $V_{T_{x}}$ is the transmitter linear velocity in m/s, $res_{i}$ is the image resolution on the *i* axis in pixels, $d_{T_{x}R_{x}}$ is the distance between the transmitter and receiver in m, and $FoV_{i}$ is the field of view on the *i* axis in degrees.

Within this range of only 5 pixels, no significant differences are observed regarding the temporal evolution of the scale and centrality of the ROI delivered by the tracking algorithm for the lateral and diagonal movements. Nevertheless, different motion speeds would affect the frontal movement results, where the transmitter changes its projected area over the image. Therefore, the speed of the transmitter is examined for the frontal case.

On the other hand, the preliminary results of the tracking algorithms are detailed in Table 3. In order to obtain the FPS values, an average of five measurements was taken for each case.

From Table 3, it can be summarized that the tracking algorithms that present scalability are: TLD, median flow, and CSRT. The one that presents a more desirable average FPS value is the median flow, with 221 FPS. Therefore, it can be concluded a priori that the optimal tracking algorithm under these selection criteria is the median flow. The processing FPS obtained for the tracking algorithms is considerably higher than the discovery algorithm (221 FPS for tracking and 10–11 FPS for discovery). This allows the system to reduce its computational load and reserve resources to accommodate faster image acquisition rates. Furthermore, it facilitates the deployment of the receiver system in a large number of devices with low computing capacity.

Based on these results, the study of the K parameters will be focused exclusively on the median flow tracking algorithm. The results, divided into lateral, diagonal, and frontal movements, are presented and discussed below. Finally, the results for the Student’s *t*-test are presented.

#### 4.2.1. Lateral Movement

**Figure 9:**
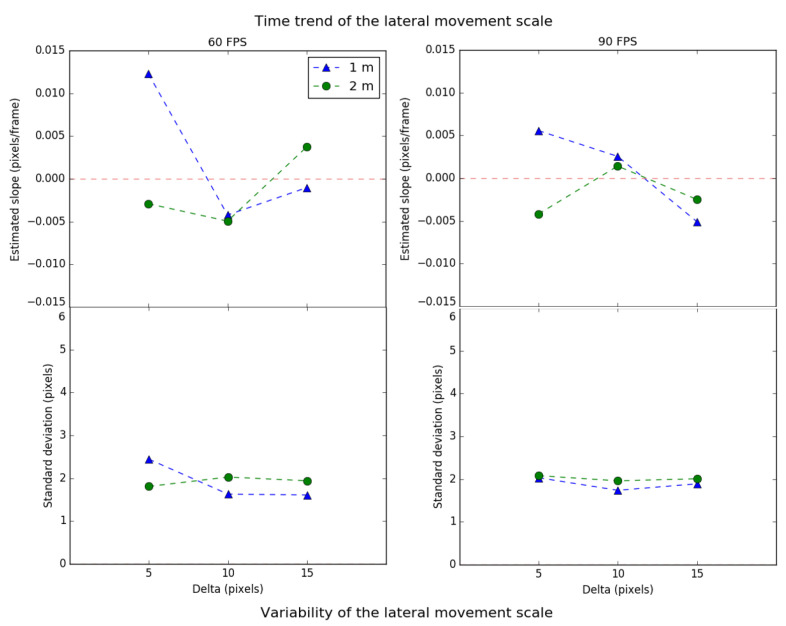
Time trend and variability of the scale for lateral movement.

**Figure 10:**
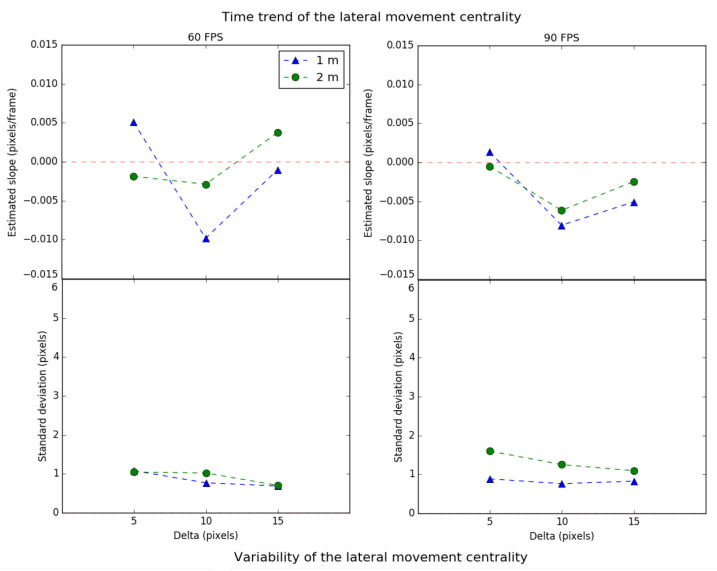
Time trend and variability of the centrality for lateral movement.

In Figure 9, the upper part shows the time trend of the lateral movement scale, while the lower part shows its variability. The left graph shows the values for a framerate of 60 FPS, for each delta, at 1 m (triangle) and 2 m (circle). The right graph shows the same parameters for 90 FPS. On the other hand, Figure 10 presents the lateral movement centrality values in the same format.

Regarding the time trend of the scale in the lateral movement, it can be observed that, in most cases, the values tend to zero, which means that the system behaves in a relatively stable way. The scale’s trend deviation meets a similar behavior regardless of the chosen starting delta (each value is close to 2 pixels). This indicates that the system’s lateral movement performance is not significantly affected by the parameters of distance, delta, and FPS. Since the trend deviation is relatively low, the selected delta of 15 pixels would prevent the tracking from losing the source for a more extended period, since the time trend scale is closer to zero for both distances.

Regarding the centrality’s time trend in the lateral movement, it is also observed that there is independence between the experiment’s parameters and the performance. In addition, the values for the centrality are lower than in the scale. This implies that the tracking, for the lateral movement, is more vulnerable to the scale effects than centrality.

Finally, it can be observed that there is a relationship between the scale and the centrality. If the first is positive, the second is also positive, and vice versa. Consequently, when the tracking decreases in area, the ROI implicitly becomes more centered. Otherwise, when the ROI increases in area, it becomes more eccentric concerning the transmitting source. The reason behind this is that the tracking algorithm is less effective in locating the transmitting source within a greater area.

#### 4.2.2. Diagonal Movement

**Figure 11:**
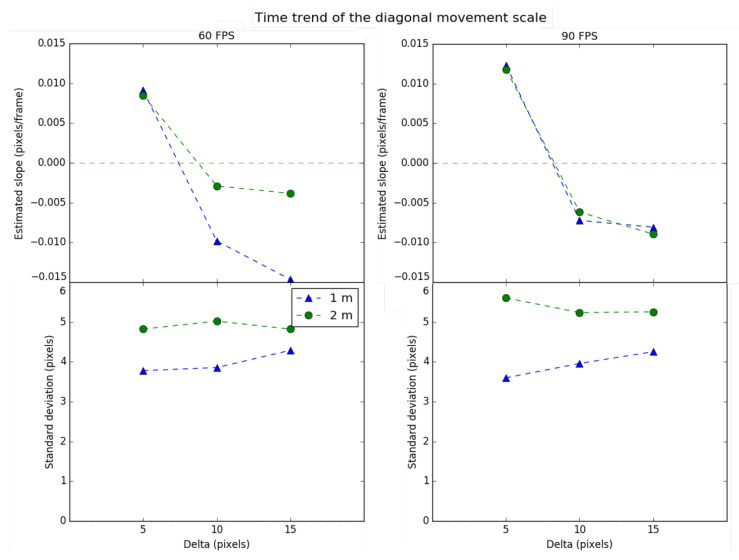
Time trend and variability of the scale for diagonal movement.

**Figure 12:**
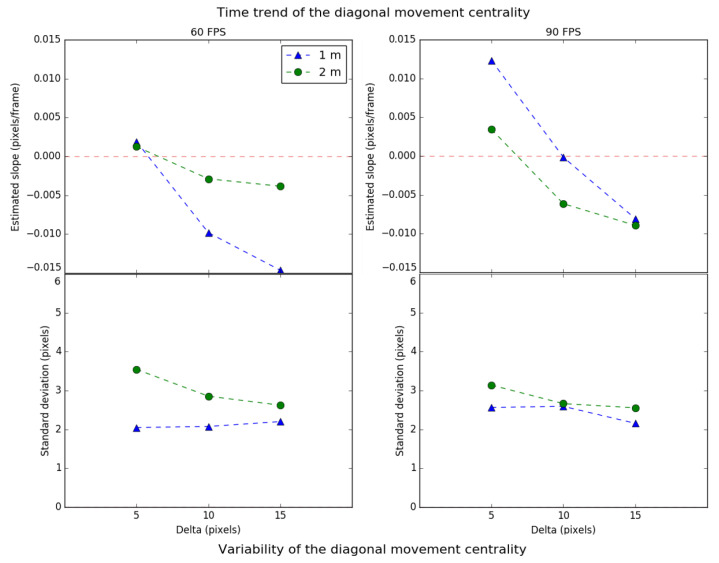
Time trend and variability of the centrality for diagonal movement.

The scale and centrality values of the diagonal movement are shown in Figure 11 and Figure 12 respectively, following the same format as the lateral movement.

Regarding the time trend of the scale, it is observed that the value of delta equal to 10 pixels presents the most stable cases. When the initial ROI area is the minimum selected (delta equals 5 pixels), the tracking algorithm has no margin of freedom, and, over time, the system’s performance deteriorates. Otherwise, when the initial ROI area is the maximum selected (delta equals 15 pixels), the algorithm cannot correctly track the light source, generally reducing the ROI area until the source falls outside. Therefore, there is a trade-off delta value for which the scale trend is minimal. Highlight that in this kind of movement, a more significant number of cases has negative slopes for the scale trend.

Regarding the time trend of centrality, it is observed that it increases as the delta decreases. This suggests that when a greater area is selected, the more eccentric the tracking will become over time because the algorithm cannot place the transmitter within the ROI correctly. From the point of view of centrality, it is better to have a low value of delta. Therefore, the diagonal movement requires a minimum value of delta for preserving centrality and an intermediate value for the scale.

In addition, it is observed that the scale and centrality trend are better for the distance of 2 m than for 1 m. This indicates that the closer the object is, the greater the pixel area projected by the transmitter in the image, increasing the probability of deterioration of the scale and centrality for the same speed. On the other hand, trend deviations for scale and centrality decrease as the distance increases, which means that they vary more softly between consecutive frames.

Finally, as a summary result, the diagonal has a worse performance compared to lateral movement. The tracking ROI contracts or expands at higher speed and variability and is quickly decentralized. Therefore, it can be concluded that the diagonal movement implies a shorter return time to the discovery state.

#### 4.2.3. Frontal Movement

**Figure 13:**
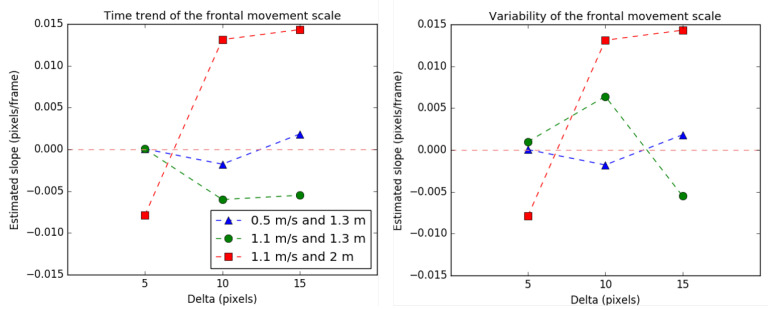
Time trend and variability of the scale for the frontal movement.

The scale values for the frontal movement are shown in Figure 13, following the same format stated in previous cases.

In this case, the scale trend generally increases with the delta. This indicates that the ROI tends to expand over time. Therefore, lower deltas reduce this unwanted effect. The independent analysis for each curve is detailed below.

In the case of transmitter motion 0.5 m/s and starting distance of 1.3 m, the time trend for the scale is the lowest for all the cases. This is because the transmitter moves with the minimum selected speed. It can be concluded that the lower the transmitter’s speed, the better the behavior of the system, as expected. In the case of transmitter motion 1.1 m/s and starting distance of 1.3 m, the time trend for the scale is greater than the previous case because of the transmitter’s speed. The higher the speed, the greater the relative motion between frames, making the tracking algorithm more susceptible to errors.

However, for the same speed but increased distance ( 2 m), in the last case, the scale trend increases considerably. This suggests that the greater the distance between transmitter and receiver, the smaller the transmitter’s pixel projection within the image, making the tracking more prone to errors.

Finally, the centrality does not deteriorate significantly over time, because the transmitter remains centered during the experiment.

#### 4.2.4. Student’s *t*-Test

About the Student’s *t*-test, the Table 4, Table 5 and Table 6 show the test results for each type of movement. Mention that “R” refers to the null hypothesis, while “NR” does not reject the null hypothesis.

It is observed that, in 91% of the cases, the null hypothesis is rejected, which implies that the system must return to the discovery regularly. The temporary deterioration of the tracking ROI is an unwanted but unavoidable effect. Furthermore, the rejection of the null hypothesis, together with the residual values following a Gaussian distribution, indicates that, with the data obtained, the use of linear regression is appropriate.

On the other hand, there are 3 of the 33 cases in which the null hypothesis is not rejected. That implies, a priori, that in these cases, the slope is zero, the tracking remains constant, and there is no need to return to the discovery. However, it should be noted that, with the data obtained, no strong relationship can be found between the parameters of the cases of non-rejection, so no parameter can be highlighted that supports this behavior.

## 5. Conclusions

In this work, an OCC receiver architecture based on discovery and tracking algorithms has been proposed. Incorporating a tracking functional block in the OCC receivers’ data acquisition pipeline considerably decreases the computation time, increasing the processing rate, and therefore the achievable data rate. On the other hand, the discovery algorithm proposed based on proposal generation algorithms and a low setting exposure time eases the detection of legitimate OCC transmitter sources, with recall greater than 99% under laboratory conditions. A novel experimental setup to evaluate the performance of the discovery and tracking algorithm is presented. It is based on evaluating the temporal evolution of the margin size between the expected and tracking delivered ROI. This analysis is based on the mean and standard deviation of the margin for all the ROI sides. This performance was evaluated for different distances, transmitter movements and speeds, and camera framerates.

In the case of the discovery algorithm, the execution time of approximately 96 ms and recall around 99% are measured, concluding that the performance is independent of the experimental conditions.

Regarding the tracking algorithms, the best-suited algorithm based on scalability support and execution time is the median flow. It allows processing rates of 221 FPS. Therefore, the combination of discovery and tracking algorithms improves the total processing frame rate of the system. As an example, considering that after two seconds of source tracking, the system returns to the discovery state, there is a processing improvement of approximately 2100% compared to the system performance, if only the discovery algorithm were used for source detection in each frame.

In addition, different movements were analyzed over time, concluding that the lateral movement presents better temporal behavior than the diagonal and frontal movement.

In all cases, it has been demonstrated that increasing the ROI that initializes the tracking algorithm by a delta factor can be effective for preventing losing the transmitter earlier, hence increasing the return-to-discovery time. For each case, there is a delta that optimizes the tracking performance. In the diagonal movement, as the delta value increases, the tracking algorithm reduces the ROI area while centering the source, causing the transmitter projection to fall outside after some time. In frontal movement, transmitter speed and distance play an important role in tracking performance. Transmitters that move further away tend to vary their projection in the image considerably with time, which causes the tracking algorithm to not easily adapt to this contraction.

Finally, the detection happens concurrently with the data transmission, independent of the encoder and modulator strategies. This is demonstrated after analyzing the detection behavior alongside random data transmissions.

## Figures and Tables

**Table 1:** Tracking algorithms.

Algorithm	Description
BOOSTING [33]	Based on the AdaBoost supervised classifier.
MIL [34]	Like the BOOSTING algorithm, instead of classifying GSRs, it classifies with a neighborhood adjacent to the object.
KFC [35]	From the neighborhoods described in the MIL, the overlapping areas are studied.
TLD [36]	Divided into three stages: tracking, in charge of tracking; detection, the object is studied, and the tracking is corrected; and learning, estimating the errors of the detector and updating it.
Median Flow [37]	Studies the temporal coherence of the trajectory; that is, it studies how a point’s trajectory advances forward and backward in time. For this reason, the median flow algorithm first tracks a point forward in time. Then, with the final position of the point, the trajectory backward in time is obtained. Finally, the difference between paths is obtained, and if they differ significantly, the forward path is discarded because it is considered wrong. Otherwise, the ROI is returned where the tracked object is likely to be.

**Table 2:** Experiment’s key parameters.

Parameter	Values
Receiver	
Camera model	PiCamera v.2
Image sensor	Sony IMX219
Image resolution [px]	640 × 480
Aperture lens	f/2
Sampling time, $t_{s}$ [µs]	18.904
Horizontal field of view, [ ${}^{\circ }$]	62.2
Vertical field of view, [ ${}^{\circ }$]	48.8
Recording time [*s*]	10.0
Exposure time, [µs]	85.0
Transmitter	
Matrix LED size [LEDs]	5 × 5
Distances between LEDs [cm]	1.4 c m
LED model	Addressable RGB APA102C
Configuration	
Types of movements	Lateral, diagonal, frontal
Distance [m]	1.3, 2
Lineal speed [m/s]	0.5, 1.1
Frames per second [FPS]	60, 90
Transmitter data hold time	2/FPS

**Table 3:** Analysis table for tracking algorithms.

Algorithm	Average FPS	Scalability
Boosting	30	No
MIL	17	No
KCF	83	No
TLD	28	Yes
Median Flow	221	Yes
Mosse	761	No
CSRT	28	Yes

**Table 4:** Student’s *t*-test for lateral movement.

		Delta		
Distance	FPS	5	10	15
2 m	60	R	R	R
	90	R	R	R
1.3 m	60	R	R	NR
	90	R	R	R

**Table 5:** Student’s *t*-test for diagonal movement.

		Delta		
Distance	FPS	5	10	15
2 m	60	R	NR	R
	90	R	R	R
1.3 m	60	R	R	R
	90	R	R	R

**Table 6:** Student’s *t*-test for frontal movement.

	Delta		
	5	10	15
0.5m/s and 1.3 m	R	NR	R
1.1m/s and 1.3 m	R	R	R
1.1m/s and 2 m	R	R	R
